# Barriers and strategies to successful tuberculosis treatment in a high-burden tuberculosis setting: a qualitative study from the patient’s perspective

**DOI:** 10.1186/s12889-021-12005-y

**Published:** 2021-10-21

**Authors:** Ivan S. Pradipta, Lusiana R. Idrus, Ari Probandari, Bony W. Lestari, Ajeng Diantini, Jan-Willem C. Alffenaar, Eelko Hak

**Affiliations:** 1 Groningen Research Institute of Pharmacy, Unit of Pharmaco-Therapy, -Epidemiology and -Economics (PTE2), University of Groningen, Antonius Deusinglaan 1, 9713 AV Groningen, The Netherlands; 2 Department of Pharmacology and Clinical Pharmacy, Faculty of Pharmacy, Universitas Padjadjaran, Bandung, Indonesia; 3 Center of Excellence in Higher Education for Pharmaceutical Care Innovation, Universitas Padjadjaran, Bandung, Indonesia; 4 Bekasi General Hospital, West Java Local Government, Bekasi, Indonesia; 5 Department of Public Health, Faculty of Medicine, Universitas Sebelas Maret, Surakarta, Indonesia; 6 Disease Control Research Group, Faculty of Medicine, Universitas Sebelas Maret, Surakarta, Indonesia; 7 Department of Public Health, Faculty of Medicine Universitas Padjadjaran, Bandung, Indonesia; 8 Department of Internal Medicine, Radboud Institute for Health Sciences, Radboud University Medical Center, Nijmegen, The Netherlands; 9 Department of Clinical Pharmacy and Pharmacology, University Medical Centrum Groningen, Groningen, The Netherlands; 10 Faculty of Medicine and Health, School of Pharmacy, University of Sydney, Sydney, Australia; 11 Werstmead Hospital, Sydney, Australia

**Keywords:** Tuberculosis, Indonesia, West Java, Tuberculosis intervention, patient’s problems

## Abstract

**Background:**

Previously treated tuberculosis (TB) patients are a widely reported risk factor for multidrug-resistant tuberculosis. Identifying patients’ problems during treatment is necessary to control TB, especially in a high-burden setting. We therefore explored barriers to successful TB treatment from the patients’ perspective, aiming to identify potential patient-centred care strategies to improve TB treatment outcome in Indonesia.

**Methods:**

A qualitative study was conducted in a province of Indonesia with high TB prevalence. Participants from various backgrounds (i.e., TB patients, physicians, nurses, pharmacists, TB activist, TB programmers at the district and primary care levels) were subject to in-depth interviews and focus group discussions (FGDs). All interviews and FGDs were transcribed verbatim from audio and visual recordings and the respective transcriptions were used for data analysis. Barriers were constructed by interpreting the codes’ pattern and co-occurrence. The information’s trustworthiness and credibility were established using information saturation, participant validation and triangulation approaches. Data were inductively analysed using the Atlas.ti 8.4 software and reported following the COREQ 32-items.

**Results:**

We interviewed 63 of the 66 pre-defined participants and identified 15 barriers. The barriers were classified into three themes, i.e., socio-demography and economy; knowledge and perception and TB treatment. Since the barriers can be interrelated, we determined five main barriers across all barrier themes, i.e., lack of TB knowledge, stigmatisation, long distance to the health facility, adverse drug reaction and loss of household income.

**Conclusion:**

The main treatment barriers can be considered to strengthen patient-centred care for TB patients in Indonesia. A multi-component approach including TB patients, healthcare providers, broad community and policy makers is required to improve TB treatment success.

**Supplementary Information:**

The online version contains supplementary material available at 10.1186/s12889-021-12005-y.

## Introduction

According to the World Health Organisation (WHO), tuberculosis (TB) is one of the top 10 causes of death and the leading cause of single infection [1]. This problem has become more complex when drug-resistant TB (DR-TB) pathogens, the TB pathogens resistant to one or more anti-tuberculosis drugs, emerge in every setting. Previous studies have shown that migration from high- to low-TB prevalence countries contributed to TB cases in the low-TB prevalence countries [2–4]. Therefore, identification of TB problems and strategies in high-prevalence countries are essential to control TB case at the global level.

With a total population of 270 million people, Indonesia is globally ranked second regarding TB prevalence and one of the top-10 countries with the highest prevalence of multidrug-resistant tuberculosis (MDR-TB) [1]. About 845,000 people in Indonesia contracted TB with 92,000 people dying from the disease and an estimated 24,000 becoming MDR-TB/ Rifampicin Resistant-TB (RR-TB) patients [1]. DR-TB became an important issue in Indonesia since its associated financial burden can reach USD 2342 per MDR-TB patient [5]. Generally, Indonesia’s reported economic TB burden is extremely high [6], with an estimated out-of-pocket health expenditure of about 48% [7].

TB’s management is an essential disease control factor. A global meta-analysis reported that previously treated TB patients were more prone to develop MDR-TB [8]. This finding is especially relevant for high-burden countries such as Indonesia, where problems affecting successful TB treatment should be a cause of concern.

The qualitative study is an effective approach to identify people’s experience, behaviour and perception that cannot be easily measured by pre-determined information from previous studies [9]. The qualitative studies looking into the barriers to successful TB treatment from the patient’s perspective in Indonesia have been published [10–12]. However, to date, no previous studies included professionals involved in the management of TB in assessing treatment barrier from the patient perspective and have either analysed the barriers to identify its core. Moreover, since the barriers can change over time, qualitative studies must be periodically repeated to provide updated information to TB stakeholders, including policy makers, healthcare providers, TB patients and the wide community. We therefore explored and analysed the main barriers to successful TB treatment from the patients’ perspective, aiming to identify potential patient-centred strategies to improve TB treatment outcome in Indonesia.

## Methods

### Context and setting

In Indonesia, tuberculosis (TB) care is managed both by the public and private health sectors, accounting from primary to tertiary healthcare facilities. The Community Health Centre (CHC), designated as ‘*Puskesmas*’, is a backbone TB care facility established as the primary public health sector at the sub-district level. Managed by the local government, CHCs have the responsibility to identify, notify and monitor TB patients within their specific area. TB care is also supported by referral hospitals, although not all referral hospitals have the facilities to support MDR-TB care. MDR-TB is managed in several centralised hospitals in Indonesia. Therefore, the prevalence and remoteness of TB have been taken into account for the selection of study sites, as they represent the complexity of TB problems and facilities in Indonesia.

West Java, an Indonesian province with 48,684,000 inhabitants in 2018 [13], was selected as the study location. In 2018 West Java had the third-highest TB prevalence in Indonesia [14]. Two districts of West Java province were selected because of their remoteness. A district located on the border with Jakarta (capital of Indonesia) was selected to represent the urban area, while the rural area was represented by a district located about 300 km from Jakarta.

### Study design

A qualitative study was performed to interpret the individuals’ common meanings of their life experiences. Within this framework, researchers attempted to understand the essence of the patients’ experience in terms of barrier to TB treatment, based on the perspective of participants from various backgrounds [9].

Purposive sampling was used to select participants with different background, age, gender, remoteness and TB experience. Sixty-six subjects were pre-defined as participants, including TB patients and non-TB patients (i.e., physicians, nurses, pharmacists, TB activist and TB programmers at the district and CHC levels). Since this study investigated the barriers to TB treatment, patients potentially experiencing problems during TB treatment (e.g., lost to follow-up/ treatment failed/ no smear sputum conversion from the first TB regimen) were selected. Thus, the inclusion criteria were TB patients being treated with a category II or MDR-TB regimen, for at least 2 months. To enrich and validate the data obtained from TB patients, we included representatives involved in the management of TB who could provide information on TB treatment. Subjects with a minimum of 6 months of experience related to TB treatment were included as the criteria of “non-TB patient” participants. Researchers and participants had no prior relationship. Participants were fully aware that the study aimed at improving TB healthcare services in Indonesia.

Interviews were conducted by two researchers: ISP (male) and LRI (female), both are PhD students with a clinical pharmacy background and interested in tuberculosis research. ISP has received both quantitative and qualitative research training and conducted public health studies involving in-depth interviews, focus group discussions (FGDs) and observational studies.

All participants provided informed consent. The informed consent form was sent to the participants at least 1 week in advance, allowing sufficient time to decide whether to join the study. All interviews were designed as face-to-face in form of in-depth interviews (IDIs) and FGDs. TB patients underwent IDIs for convenience to prevent the risk of stigma and potential sensitive as well as confidential issues that will be shared by TB patients. Similarly, key persons from the local government and non-governmental organisations; and medical specialists were also interviewed in form of IDIs due to time availability. On the other hand, FGDs were employed for healthcare providers (i.e., general practitioners, pharmacists, and TB programmers/ nurses at the CHC level) to obtain a range of view and understand the treatment barriers of TB patients in a population.

Only participant and interviewer who were in the interview location for IDIs. However, we included an interview assistant to note the essential topics during FGDs. Each interview started with general questions using the Indonesian language (Bahasa), then the interviewer explored and expanded the information based on pre-established research questions. For TB patients, the general questions included ‘Can you tell me about your experience as a TB patient? (What, where, why and how)’ and ‘What are the main TB problems that you have faced before and after your TB treatment?’. In contrast, for non-TB participants, general questions included ‘What are your activities in TB management’; ‘what are the main TB treatment problems from the patient’s and healthcare providers’ perspectives?’. The interview followed several steps according to the interview guide shown in Additional file 1**.**

### Information’s trustworthiness and credibility

Information saturation was defined as no emergence of new information relevant to the study’s objective and was used to determine the final number of participants during the interview process. Participants were re-interviewed whenever further clarifications were needed. IDIs were audio-recorded, whereas FGDs were audio-visually recorded to recognise participant statements within the data analysis. At the end of each interview, interviewers discussed the findings and made notes on essential information requiring further exploration. Information cross-validation among participants was employed to enhance information trustworthiness. Other sources, such as documents, regulations and standard operating procedures, were also explored to increase information credibility. To validate the interview, preserve research ethics, and empower the participants, we provided an opportunity for the participants to read and approve the interview’s transcript without any pressure and guidance to agree with specific meanings. The literacy level of the participant was ascertained before and during the interview. Inability to read and understand the majority question, term and discussion context during the interview process were signs for potential low literacy level. Interviewer-related bias was addressed by continuously discussing and negotiating the content of keywords, broader concepts and units of meanings among the research team.

### Data analysis

All interviews in the original language (Bahasa) were transcribed verbatim from audio and visual recordings. All transcripts left out any participant’s identification and were transferred to the Atlas ti (8.4) software for data analysis. The formal analysis process involved four main stages: familiarisation, thematic framework identification, codification and interpretation. Familiarisation aimed to identify a general thematic framework, by creating a segmentation of the transcript’s meaning unit. The meaning unit was identified by the transcript’s sentences that related to the study objective. Once the general thematic framework was created, coding was performed. ISP inductively coded the transcript’s information and the codes were discussed with the second coder (LRI). Identified codes were classified into themes/sub-themes according to the created general thematic. Field notes were also reviewed during the coding and themes/sub-themes development process. Data interpretation was performed by analysing the code’s pattern among the participants. Potential relationships across the codes were investigated through co-occurring codes, which overlapped in a meaning unit. Emerged codes, themes and sub-themes were translated into English (ISP, LR) and reviewed by other researchers (AP, BWL, AD, JWA, EH) through peer debriefing. Disagreements were resolved among research team members, through continuous content discussions and negotiations based on the transcripts and field notes. The consolidated criteria for reporting qualitative studies (COREQ)-32 items [15] was followed for reporting this study. The example of coding process and COREQ-32 item checklist are presented in Additional files 2 and 3**,** respectively.

## Results

We obtained consent from 63 of the 66 pre-defined participants in Bahasa Indonesia. No other local languages have been used since all participants can communicate with Bahasa Indonesia both in oral and written communication. Among the pre-defined participants, four (2 general practitioners, 1 TB nurse and 1 TB patient) did not participate in the interview process. Lack of back fill for daily clinical work has been the reason of three healthcare staffs in the absence of interview, while 1 TB patient could not be contacted during the research period. We performed a data follow-up for an additional participant (the wife of a TB participant), following her provision of informed consent for further clarification and information exploration by phone. Finally, the study included data from 63 participants and information saturation was achieved from the interviews. The in-depth interviews were conducted for 19 interviews of 19 participants, while FGDs were conducted for nine groups of 44 participants. The participants’ characteristics are presented in Table 1.

**Table 1:** Characteristics of the participants (*N* = 63)

No	Characteristics	Number
1	Background of the participants (n, %)	
	*TB patients*	
	Non-MDR-TB patient	5 (7.9)
	MDR-TB patient	4 (6.3)
	*Health care workers at the community setting*	
	Physician of the CHC	8 (12.7)
	Nurse / TB programmer of the CHC level	10 (15.9)
	Pharmacist of the CHC	10 (15.9)
	Community pharmacist	13 (20.6)
	*Health care workers at the hospital setting*	
	TB nurse	1 (1.6)
	Pharmacist	1 (1.6)
	Pulmonologist	1 (1.6)
	Internist	1 (1.6)
	*Others*	
	Government sector	5 (8.0)
	Tuberculosis activist	1 (1.6)
	Patient’s family	1 (1.6)
	Profesional organization at the district level	2 (3.2)
2	Male gender (n, %)^a^	16 (25.4)
3	Age, in year (mean; min-max)	40.38; 16–66
4	Experience in TB, in month (mean; min-max)	89.46; 6–348
5	The number of participant (n, %)	
	In-depth Interview	19 (30.2)
	Focus Group Discussion	44 (69.8)
6	Duration of the interview, in minute (mean; min-max)	80.80; 4–124
7	Area (n, %)	
	Rural	29 (46)
	Urban	34 (54)
8	Interview’s location (n, %)	
	Health district office	26 (41.3)
	Community health service	21 (33.3)
	Professional organization’s office	9 (14.3)
	Hospital	5 (7.9)
	Home	1 (1.6)
	Non-Governmental Organization’ office	1 (1.6)

^a^Number of male: non-MDR-TB patient (3); MDR-TB patient (2); Physician of the CHC (1); Nurse / TB programmer of the CHC level (1); Pharmacist of the CHC (0); Community pharmacist (6); Hospital TB nurse (0); Hospital pharmacist (0); Pulmonologist (0); Internist (1); Government sector (1); Tuberculosis activist (0); Patient’s family (0); Professional organization at the district level (1)

A total of 184 meaning units were gathered in the analysis. The highest number of codes was produced by the group of TB patients. The meaning units were converted into codes and classified into themes and sub-themes. To enhance the findings’ credibility, all emerged codes were confirmed by at least three participant sources, except for the treatment duration code, which was gathered from only two participants—the TB programmer at the CHC level and the hospital’s nurse. However, upon reviewing the national guideline [16] we found that the minimum treatment duration for active TB patients is relatively long (6 months). Therefore, the information gathered from the two participants during the interview was supported by the national guideline. The duration of TB treatment was determined as a patient’s barrier to treatment success. The code pattern can be seen in Additional file 4**.**

**Fig. 1:**
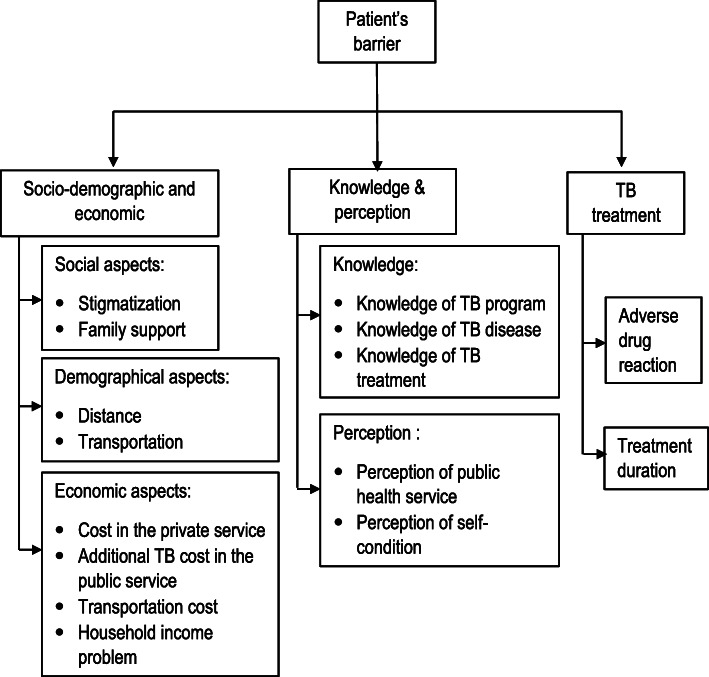
Barriers to successful TB treatment from the patient perspective

We identified three themes: (1) socio-demographic and economic; (2) knowledge and perception; and (3) TB treatment. The socio-demographic and economic theme was constructed from the socio-demography and economic aspects. We constructed the knowledge and perception theme from TB knowledge and perception aspects. Lastly, TB treatment theme was constructed from codes related to TB treatment, such as adverse drug reaction and treatment duration. The classification of themes, sub-themes and codes is described in Fig. 1**.**

### Social aspects—stigmatisation and lack of family support

Stigmatisation of TB patients was identified from the community, in that the patients felt that the community disapproved of them and feared close contact with them.

‘*If I meet people, they look afraid of me*.’—Male TB patient, 33 years old.

In addition, perceived stigmatisation arose not only from the general community but also from close family and healthcare providers.

‘*There was a man who rejected his wife due to TB, which may have been incited by his family. The wife was expelled by her mother-in-law, and she was finally divorced*.’—Female TB activist, 32 years old.

‘*The stigma exists even in the CHC from healthcare providers. They do not want to inject the medicine. It causes inconvenience and disgrace to the patients*.’— Female General Practitioner’ (GP) CHC, 41 years old.

Participants receiving TB treatment during the study period reported lack of spouse support. For example, one patient was left alone by his wife due to his poor condition. He was asked to move back to his hometown and live separately with his children.

‘*My wife assumed that I could not be cured because of the disease severity. That is why she left me.*’—Male MDR-TB patient, 29 years old.

Lack of spouse support was confirmed by the CHC TB programmer upon observing a lost to follow-up patient. This barrier was identified from a husband who prevented his wife from having further TB treatment.

‘*During my field observations, a husband said that his wife was already cured and she did not have to come to CHC anymore*.’—Female nurse/ CHC TB programmer, 40 years old.

### Demographic aspects—long distance and difficulties in reaching public transportation

Accessibility to the public health facility was the main patient’s barrier in the demographical aspects. Long distance to the public health service added another burden to TB patients while they were receiving a regular injection of category II or MDR-TB regimen.

‘*The distance from my home to this Puskesmas (community health service) is long. I cannot afford the cost of a taxi bike every day*.’—Male TB patient, 33 years old.

MDR-TB centre unavailability at the district level makes it difficult to access a public health facility. In the study, an MDR-TB patient experienced difficulty to receive diagnosis and treatment for lack of a proper facility. It would take him around 4 h to reach the centre using public transportation and he had to spend his own money.

‘*Because we want to get fast action, I went to the MDR-TB centre using public transportation. I stayed there for two days because of the distance.*’—Male MDR-TB patient, 33 years old.

### Economic aspects—cost of the private/public health service, transportation cost and loss of household income

Although the government has claimed free TB service, TB patients who visit private health sectors have to pay for TB care, including diagnosis and treatment. Due to lack of information about free TB programs in the public health sector, some patients with poor living condition are required to spend their own money to purchase medications in the private health sector.

‘*Some medicines must be taken regularly. I bought the medicine in a pharmacy every month.*’—Male MDR-TB patient, 33 years old.

Demographical problems that are shown in the participant’s experiences affect the cost of treatment. Since patients within the category II regimen must receive injectable medicines, regular visits to the health facility are required. Unfortunately, a transportation subsidy was only provided to MDR-TB patients, which means no transportation subsidy for patients with category II regimen.

‘*No transportation subsidies are available for the patient in the category II regimen. They should come to CHC every day. The problem is when we have patients with low economic level.*’—Female CHC’s GP, 50 years old.

Our study revealed that TB patients who visit a public health service spent an additional cost. Participants expressed their experience in spending their own money in a public health service. One of them told us that an additional cost was needed for injectable medicines.

‘*Although in this Community Health Centre the medical examination and medicine were free, I spent my own money for the injectable medicine using healthcare staff from another CHC*.’—Male TB patient, 54 years old.

‘*The problem is when the patients do not have a health insurance. Sometimes they have to be referred to a hospital for a paid chest X-Ray*.’—Female CHC’s GP, 39 years old.

Furthermore, we identified a barrier related to TB patients’ household income. Patients must do their work activities to continue their life. Some TB patients stopped treatment because they had to work to fulfil their household income.

‘*MDR-TB patients, especially men, face extraordinary challenges. As head of the family, they should be the backbone of family income. Although we educated them, most patients had a defaulted treatment*’—Female TB nurse, 31 years old.

### **Knowledge aspects**—k**nowledge about the TB program, disease and treatment**

Insufficient knowledge on the implementation of the free TB program was explicitly identified in TB patients who reached TB care through private health services. The problem arose when TB patients started the treatment in the private health service. They could not afford the medical expenses until the end of the treatment, which led to its discontinuation.

‘*Initially, treatment lasted for six months and was mandatory. However, I stopped the medicine because I did not know that the healthcare service was free of charge. I stopped the treatment because of the cost.*’—Male TB patient, 33 years old.

Adding to these problems, lack of knowledge on TB programmes was worsened by the lack of coordination between the private and public health service facilities. Some private health sectors have low motivation to refer TB patients to a public health facility. We identified resistance from the private health providers to transfer a TB patient to the CHC. Different ingredients and different medicine forms were indicated as reasons to hold TB patients treated in the private service. According to the National guideline, TB regimen was standardised and provided in public health facilities.

‘*In the middle of the treatment period, I asked the private doctor to provide me a referral letter to a Puskesmas. The doctor answered that my husband could not move to a CHC, since he was being administered a specially compounded medicine made by the doctor himself*.’—Wife of TB patient, 45 years old.

An MDR-TB patient who delayed treatment, indicated lack of knowledge and awareness about TB as the cause for delayed treatment.

‘*I ignored the signs for six months until I read about TB. After that, I decided to get a medical examination.*’—Male MDR-TB patient, 33 years old.

The study also revealed that participants tried to self-medicate with herbal medicine and to purchase anti-TB drugs at the private pharmacies without proper medical examination.

‘*Because I felt my symptom was lessen using the previously prescribed medicines, I decided to keep the packaging of the medicines. Whenever I experience the same symptoms, I bought package and buy the medicines directly*.’—Male TB patient, 33 years old.

‘*A patient said that ‘I was treated in my village’, but TB treatment only lasted for one week because the patient experienced itchiness. The patient then continued treatment using herbal medicines*’—Female CHC’s GP, 39 years old.

Lack of knowledge regarding adverse reactions and treatment duration were identified by participants. Our study successfully detected a patient who was advised to stop the medicine due to treatment side effects.

‘*My family advised me to stop the medicine, due to adverse drug reactions. They said that the medicine worsened my condition.*’—Male MDR-TB patient, 29 years old.

### Perception—perception of public health services and self-condition

There was a negative image regarding the quality of TB medicine in CHC. Some patients believed that qualified medicine should be expensive and not free.

‘*There are rich people who do not want to go to CHC because they assume that free of charge medicines have poor quality*.’—Female TB coordinator, 43 years old.

Our observations demonstrated that the CHCs have provided a special line for persons suspected of having TB or persons diagnosed with TB to shorten the waiting time and control disease contamination. As a result, the patients can be directly examined by a physician. However, there were some negative comments about the public health service. One interviewee argued that he would not go to the public health service due to his bad experience about waiting time for getting the medical examination.

‘ *I asked my husband to visit the nearest CHC, but he said no, because of the long queue of patients*.’—wife of TB patient, 45 years old.

Another reported problem regarded physician preference. Some patients prefer to be examined and treated by a famous private physician in their area than by a physician in the public health service.

‘*They may have a suggestive (placebo) effect when they go to a famous physician or private health facility instead of CHC, so they do not choose CHC*.’—Female TB programmer/Nurse at CHC, 31 years old.

Psychological problems emerged in TB patients due to a negative perception of their condition. They felt disgrace, hopelessness and rejection due to their health condition. Another identified story regarded the rejection of a wealthy patient for assuming that TB only affects people with poor living condition.

‘*Because of this disease, I fell in disgrace with my neighbours.*’—Female TB patient, 41 years old.

‘*I have felt in that position, and I felt tired. It was better to die.*’—Female TB activist, 32 years old.

‘*If rich people were getting TB, they seemed to have a rejection.’*—Female Hospital pulmonologist, 45 years old.

### TB treatment—treatment duration and adverse drug reactions

As widely known, active TB patients should follow treatment for at least 6 months. Our study identified boredom and low treatment adherence. Furthermore, adverse drug reactions were highly reported from the participants, potentially leading to unsuccessful treatment.

‘*The fact that the patient got bored of taking medicines for a long time was a common problem.*’—Female TB programmer/ nurse at CHC, 31 years old.

‘*I felt a headache, dizziness, flying and I hallucinated buying a car. It was like a crazy person.’*—Female MDR-TB patient, 16 years old.

### Potential relationship

We identified several co-occurred codes in a meaning unit, indicating a potential relationship across the codes. We, therefore, analysed the barriers considering the co-occurred data. There are five main barriers identified across those codes, i.e., lack of TB knowledge, stigmatisation, long distance to the health facility, adverse drug reaction and loss of household income. Data co-occurrence and constructed barriers are presented in Table 2 and additional file 5, respectively.

**Table 2:** Co-occurrence of data and potential relationships

No	co-occurrence	Meaning unit	Interpretation
1	Lack of knowledge about TB disease AND delayed treatment	‘*I ignored the signs for 6 months until I read about TB. After that, I decided to get a medical examination*.’—MDR-TB patient, 33 years old	Lack of knowledge about TB disease is a cause of delayed treatment
2	Lack of knowledge about TB treatment AND inappropriate medicine use AND non-adherence	*’Because I felt my symptom was lessen using the previously prescribed medicines, I decided to keep the packaging of the medicines. Whenever I experience the same symptoms, I bought package and buy the medicines directly.’*—TB patient, 33 years old*.*	Lack of knowledge about TB treatment is a cause of inappropriate medicine use and non-adherence
3	Lack of knowledge about TB program AND Financial problems	‘*Initially, treatment lasted for 6 months and was mandatory. However, I stopped the medicine because I did not know that the healthcare service was free of charge. I stopped the treatment because of the cost*.’—TB patient, 33 years old.	Lak of knowledge is a cause of financial problems
4	Lack of knowledge about TB program AND Negative perception of public health service AND financial problem	‘*There are rich people who do not want to go to CHC because they assume that free of charge medicines have poor quality*.’—TB coordinator, 43 years old. ‘*I asked my husband to visit the nearest CHC, but he said no, because of the long queue of patients*.’—wife of TB patient, 45 years old.	Lack of knowledge about TB programs is a cause of negative perception about the public health service and financial problems
5	Lack of knowledge about TB disease and treatment AND negative perception of self-condition	‘*I have felt in that position, and I felt tired. It was better to die*.’—TB activist, 32 years old.	Lack of knowledge about TB disease and treatment is a cause of the negative perception of self-condition and psychological problems
6	Distance to health facility AND financial problems AND inaccessible qualified TB care	‘*The distance from my home to this Puskesmas (community health service) is long. I cannot afford the cost of a taxi bike every day.*’—TB patient 33 years old.	Long distance to a public health facility is cause of financial problems and inaccessibility to a qualified TB care
7	Stigmatisation AND isolation/discrimination AND lack of family support	‘*My wife assumed that I could not be cured because of the disease severity. That is why she left me*.’—MDR-TB patient, 29 years old. *‘There was a man who rejected his wife due to TB, which may have been incited by his family. The wife was expelled by her mother-in-law, and she was finally divorced.*’—TB activist, 32 years old.	Stigmatisation is a cause of isolation/discrimination and low family support
8	Negative perception of self-condition AND inaccessible qualified TB care	‘*Sometimes, I feel inferior and shy for going to CHC because of my disease*.’—TB patient, 33 years old, male.	A negative perception of the self-condition is cause of inaccessible qualified TB care
9	Household income AND Financial problems AND Unsuccessful TB treatment	‘*MDR-TB patients, especially men, face extraordinary challenges. As head of the family, they should be the backbone of family income. Although we educated them, most patients had a defaulted treatment*’—TB nurse, 31 years old.	Low household income due to TB is a cause of financial problems and unsuccessful treatment
10	Adverse drug reaction AND psychological problems	‘*I felt a headache, dizziness, flying and I hallucinated buying a car. It was like a crazy person*.’—MDR-TB patient, 16 years old.	ADR is a cause of psychological problems
11	Adverse drug reaction AND non-adherence and non-persistence	‘*A patient said that ‘I was treated in my village’, but TB treatment only lasted for 1 week because the patient experienced itchiness. The patient then continued treatment using herbal medicies’*—CHC’s GP, 39 years old.	ADR is a cause of non-adherence/persistence

## Discussion

In this study we highlighted the barriers to successful TB treatment from the patient’s perspective and found that they are related to three themes as follows: (1) socio-demography and economy; (2) knowledge and perception and (3) TB treatment. The socio-demography and economy theme comprises several barriers, such as stigmatisation, lack of family support, long-distance to public health service, transportation difficulties, cost of the private and public health service, cost of transportation and loss of household income. The knowledge and perception theme includes lack of knowledge about TB (i.e., TB program, diseases and treatment), negative perception of public health service and self-condition. As mentioned above, the TB treatment theme concerns the barriers of adverse drug reaction and long duration of treatment. Since the barriers can be interrelated, we identified five main barriers: lack of TB knowledge, stigmatisation, long distance to the health facility, adverse drug reaction and loss of household income.

Our results are consistent with a previous study that indicates that stigmatisation is one of the barriers experienced by TB patients in Indonesia. Watkins and Plant (2004) stated that people with TB still carry a social stigma from the community in Bali [10]. Unfortunately, the previous study did not provide details regarding the source of the stigma. We identified that the stigma originates not only from the community but also from the close family and healthcare providers in the study setting.

The stigma originating from close family generates discrimination and isolation in TB patients. In the present study, several patients reported to have been left alone by their close family, without any support in facing the disease. The issue gets more complex when stigmatisation is also identified in the community and workplace, as this can influence the patients’ ability to access qualified TB care and to generate the daily income required for survival. In addition, TB patients do not have social security in Indonesia. Although the government has announced a free TB care programme, our study demonstrated that some costs were still covered by the patients themselves. Some participants mentioned that some costs such as transportation, private services and additional services in public health facilities, had to be covered by themselves.

Our study identified a TB patient who went to a different health facility to receive injectable medicines, which was paid with his own money. Two reasons for this issue were identified: (1) Limited-service time and health care resources for TB patients in CHC, which commonly happens only twice a week, on ‘TB days’; and (2) the Existence of health condition-related stigma in health facilities.

In the present study, the occurrence of health condition-related stigma in health facilities was identified by the fear of TB felt by the healthcare staff. The staff realised that this can affect the patients’ psychological condition, leading to sub-standard of care. Previous studies showed that health condition-related stigma is a barrier to health-seeking behavior [17], engagement in care [18] and adherence to treatment [19]. Consistently, our study also identified this as a main barrier. Several aspects such as lack of family support, isolation, discrimination, psychological problems and inaccessibility to qualified TB care were observed. All contributed to unsuccessful TB treatment.

Previous studies have clarified the association between knowledge, perception and health-related behaviour [20]. We found that lack of knowledge about the national TB programme and public health facilities potentially cause a negative perception of the public health services, contributing for inaccessibility to qualified TB care, especially for people with low income. We also found that TB patients did not want to go to the public health services due to their perception about the quality of services and medicines. Another finding showed that some patients were not aware that the public health service had a free TB programme. This may lead patients with poor living condition having lost to follow-up treatment due to the cost burden because patients must spend their own money to receive TB care in private health services. Our study provided additional information from a previous study that economic burden is not only an impact of non-adherence to medication [21], but also a causal factor of medication adherence. It showed a necessity to use personalized approaches to improve medication adherence considering multifaced adherence factors in tuberculosis patients [22]. Generally, we analysed that lack of knowledge on TB and its treatment may lead to non-adherence and non-persistence, delayed treatment and a negative perception of self-condition.

Regarding physiological problems, TB patients could be also affected by adverse drug reactions and negative perception of self-condition. For example isoniazid, ethambutol, fluoroquinolones and cycloserine can induce psychiatric disorders in tuberculosis patients [23]. Moreover, the level of education can also impact the patients’ perception of their health status [24]. It can be argued that self-condition perception can be driven by the patient’s knowledge of the disease and treatment. Therefore, the patient’s knowledge has an important role in the control of physiological problems in TB patients beside of the adverse drug reaction management.

### Strength and limitations

Several limitations should be acknowledged in the present study. First, the findings were analysed by the emergence of the code from the participant’s information. Although in qualitative studies this is a generally used approach, further quantitative investigation is needed to generalize the result into the population level. Secondly, the identified barriers may not be the same in the areas that have different healthcare system, social, economic, cultural and political context. Thirdly, gender influence was not taken into account in this analysis, due to limited access to the male gender in the group of “non-TB patient” participant. However, several approaches were used in this study, such as maximising the characteristics of the participants and study location (rural vs urban); using a triangulation method and respondent validation; analysing the code pattern, data co-occurrence and information saturation; employing continuous discussions about the obtained data among the research team. We also interviewed non-TB patients to enrich information and to take the patient’s barrier seriously. Therefore, we believe that such approaches contribute to the validity and reliability of our study.

### Study implications

The main treatment barriers identified can be further investigated using the quantitative study to generalize the findings to the population level. The quantitative analysis will guide TB stakeholders to develop effective interventions for improving treatment outcome from the population perspective. However, several approaches should be considered to support patient-centred care for TB patients in Indonesia.

Educating patients, healthcare providers and the community is needed to prevent and reduce the stigma. TB patients should be counselled on how to deal with potential stigmatisation and psychological problems. Similarly, public should be educated on how to adequately act and support TB patients for treatment success. The existence of a qualified TB counsellor supported by guidance at the CHC level may help to address stigmatisation. Moreover, stigmatisation knowledge and awareness should also be improved among healthcare providers [25]. Healthcare providers should be ensured about their capability and facility to manage TB patients, since the occurrence of stigma can be generated by fear of the disease, lack of awareness, inability to manage the patient, inadequate facility and institutional procedures or practices [25–29].

We analysed that long distance to public health facilities is associated with the patient’s treatment cost burden, especially for MDR-TB patients. As described participant’s thoughts during the interview, a commitment from the central and local government to fully decentralise MDR-TB care at the district level may solve this issue. A previous meta-analysis reported that a decentralised approach was associated with higher treatment success among MDR-TB patients [30]. This problem may be further reduced by a new fully oral treatment for MDR-TB, by reducing long visit duration, patient inconvenience and unavailability of proper transportation to the referral hospital.

Regarding adverse drug effects, access to a drug consultant who supports and educates TB patients may help to minimise this issue. A reasonable approach to tackle this issue could be to involve a pharmacist for direct patient service in TB management, especially at the CHC level. As a result from the limited national guidance [16], institutions should prepare pharmacists to be involved in direct patient service [31]. Some arrangements may be required in terms of pharmacists’ involvement, such as availability, TB knowledge and specific service guidelines [32]. Pharmacists may act as treatment supporters who educate, monitor and evaluate medicine use, based on the principles of pharmaceutical care. It was previously reported that pharmacists’ direct involvement in TB patients’ management improved treatment success [33, 34].

Lastly, social protection schemes, reaching beyond direct medical costs, such as loss of household income, should be a concern for the government. This might tackle issues related to lost to follow-up treatment due to the patient’s decreasing household income. Special attention and priority should be given to patients with MDR-TB and the lowest income groups since both groups have the highest potency for economic vulnerability as a result of the disease [35].

## Conclusion

This study has identified several barriers to successful TB treatment, from the patient’s perspective, in Indonesia. The barriers were classified into three themes: (1) socio-demography and economy; (2) knowledge and perception; and (3) TB treatment. Our findings indicate that there are five main treatment barriers across those themes, i.e., lack of TB knowledge, stigmatisation, long distance to the health facility, adverse drug reaction and loss of household income. The main treatment barriers should be considered to strengthen patient-centred care for TB patients in Indonesia. A multi-component approach including TB patients, healthcare providers, broad community and policy makers is required to improve TB treatment success. The main treatment barriers identified can be further investigated using the quantitative study to generalize the findings and develop the effective intervention at the population level.

## Supplementary Information

**Additional file 1.**

**Additional file 2.**

**Additional file 3.**

**Additional file 4.**

**Additional file 5.**

## Data Availability

The data are in the form of photographs: video and audio recording; video and audio transcripts; and field notes. All the data are located in the data storage of corresponding author. The datasets generated and/or analysed during the current study are not publicly available due ethical reason but are available from the corresponding author on reasonable request considering ethical issues in the qualitative study.

## Abbreviations

WHO: World health organization
TB: Tuberculosis
DR-TB: Drug-resistant tuberculosis
MDR-TB: Multidrug-resistant tuberculosis
USD: United States dollar
CHC: Community health care center
IDIs: In-depth interviews
FGD: Focus group discussions
COREQ: The consolidated criteria for reporting qualitative studies
GP: General practitioner
