# Supplementary material for: Barriers and strategies to successful tuberculosis treatment in a high-burden tuberculosis setting: a qualitative study from the patient’s perspective
Source: BMC Public Health. 2021 Oct 21;21:1903. doi: 10.1186/s12889-021-12005-y (PMC8529853; doi:10.1186/s12889-021-12005-y)
Supplement: Supplementary file 1 — Additional file 1. [file 12889_2021_12005_MOESM1_ESM.docx]

**Additional file 1.** Guideline of the interview

There are several steps for conducting an in-depth interview:

1. Preparation
   1. Ensuring the location of FGD and tools needed (e.g., recorder, camera, stationery, mini-board, circle seating)
   2. Ensuring the criteria of the participant and number of participants
2. Introduction
   1. Giving appreciation for participation in this study
   2. Identifying name, age, and background of participant (patients/ non-patients)
   3. Explaining the study, informed consent, and purpose of the interview
   4. Informing that all interview will be confidentially recorded and used for a scientific purpose only
   5. Informing the average of interview duration max. 120 minutes
   6. Informing about how the interview will be conducted, including an emphasized statement that the participants may end the interview anytime
   7. Giving an opportunity for the questions
   8. Giving an opportunity to read and sign the consent
3. Questions

The open-ended questions will be given, and the questions will be asked about the factual condition before the opinion.

1. Closing statement
   - 1. Offering additional comment or question related to the interview or study
     2. Confirming the important notes/findings to the participant
     3. Giving a thank you statement for the participation in this study

There are several steps for conducting the focus group discussion:

1. Preparation
   1. Ensuring the location of FGD and tools needed (e.g., recorder, camera, stationery, mini-board, circle seating)
   2. Ensuring the criteria of the participant and number of participants
2. Introduction
   1. Introducing moderator and assistant moderators
   2. Explaining the background of the study and objective of the FGD
   3. Explaining about the informed consent and guideline of FGD:
   - No right answer
   - The FGD will be confidentially recorded and analyzed
   - Informing about how the interview will be conducted, including an emphasized statement that the participants may end the interview anytime
   - The participants do not need to agree with other participants but must listen respectfully to others’ views
   1. Giving an opportunity for the questions
   2. Giving an opportunity to read and sign the consent
3. Questions

The question will start with general questions: “ What are your activities in TB management”; “What are the problems of TB from the patient and healthcare providers perspectives?”

1. Closing statement
   - - - Offering additional comment or question related to the interview or study
       - Confirming the important notes/findings to the participant
       - Giving a thank you statement for the participation in this study
