# Supplementary material for: Barriers and strategies to successful tuberculosis treatment in a high-burden tuberculosis setting: a qualitative study from the patient’s perspective
Source: BMC Public Health. 2021 Oct 21;21:1903. doi: 10.1186/s12889-021-12005-y (PMC8529853; doi:10.1186/s12889-021-12005-y)
Supplement: Supplementary file 2 — Additional file 2. [file 12889_2021_12005_MOESM2_ESM.docx]

**Additional file 2**. The example of the coding process

| **Meaning unit** | **Codes** | **Sub-theme** | **Theme** |
| --- | --- | --- | --- |
| *‘The stigma exists even in the CHC from healthcare providers. They do not want to inject the medicine. It causes inconvenience and disgrace to the patients.’* | Stigmatization | Social aspect | Socio- demography and economy |
| *‘Because we want to get fast action, I went to the MDR-TB centre using public transportation. I stayed there for two days because of the distance.’* | Distance | Demographical aspects |  |
| *‘Some medicines must be taken regularly. I bought the medicine in a pharmacy every month.’* | Cost in the private service | Economical aspect |  |
| *‘Initially, treatment lasted for six months and was mandatory. However, I stopped the medicine because I did not know that the healthcare service was free of charge. I stopped the treatment because of the cost.’* | Knowledge of TB program | Knowledge | Knowledge and perception |
| *‘They may have a suggestive (placebo) effect when they go to a famous physician or private health facility instead of CHC, so they do not choose CHC.’* | Perception of the physician | Perception |  |
| *‘I felt a headache, dizziness, flying and I hallucinated buying a car. It was like a crazy person.’* | Adverse drug reaction | Adverse drug reaction | TB treatment |
| *‘The fact that the patient got bored of taking medicines for a long time was a common problem.’* | Treatment duration | Treatment duration |  |
