# Supplementary material for: Barriers and strategies to successful tuberculosis treatment in a high-burden tuberculosis setting: a qualitative study from the patient’s perspective
Source: BMC Public Health. 2021 Oct 21;21:1903. doi: 10.1186/s12889-021-12005-y (PMC8529853; doi:10.1186/s12889-021-12005-y)
Supplement: Supplementary file 4 — Additional file 4. [file 12889_2021_12005_MOESM4_ESM.pdf]

Additional file 4. Codes' pattern among the study participants

No	Codes	CHC's Physician	CHC' Pharmacist	CHC's TB Programmer / nurse	Community pharmacist	Hospital pharmacist	Hospital's nurse	Local government key persons	TB patients	TB specialist	TB Activist	Totals
1	Socio, demography, economic											
	Stigmatization	4	2	4	0	0	0	0	3	0	3	16
	Family support	0	0	1	0	0	0	1	1	0	2	5
	Distance	0	0	0	0	0	0	0	5	0	0	5
	Difficulties in public transportation	0	0	1	1	0	0	1	0	0	0	3
	Additional cost in the public services	1	0	0	0	0	0	0	1	1	0	3
	Cost for diagnosis and treatment in the private clinic or hospital	3	1	3	5	0	0	2	8	0	0	22
	Household income problem	0	0	2	0	0	0	0	0	0	1	3
	Transportation cost	1	0	0	0	0	0	1	3	0	0	5
2	Knowledge and perception											
	Lack of knowledge about TB disease	5	2	6	2	0	0	0	2	1	2	20
	Lack of knowledge about TB program	0	0	0	1	0	0	0	3	0	0	4

	Lack of knowledge about TB treatment	2	3	3	12	0	0	3	8	1	0	32
	Patient perception of public health service	1	0	0	1	0	0	1	0	0	0	3
	Patient perception of the physician or health care provider	0	0	1	3	0	0	0	0	1	0	5
	Perception on self-condition	2	0	2	2	0	0	0	3	1	4	14
3	TB treatment											
	Adverse drug reaction	5	4	8	0	0	1	2	16	3	3	42
	Long duration of treatment	0	0	1	0	0	1	0	0	0	0	2
**Total**												**184**
