# Supplementary material for: Barriers and strategies to successful tuberculosis treatment in a high-burden tuberculosis setting: a qualitative study from the patient’s perspective
Source: BMC Public Health. 2021 Oct 21;21:1903. doi: 10.1186/s12889-021-12005-y (PMC8529853; doi:10.1186/s12889-021-12005-y)
Supplement: Supplementary file 5 — Additional file 5. [file 12889_2021_12005_MOESM5_ESM.pdf]

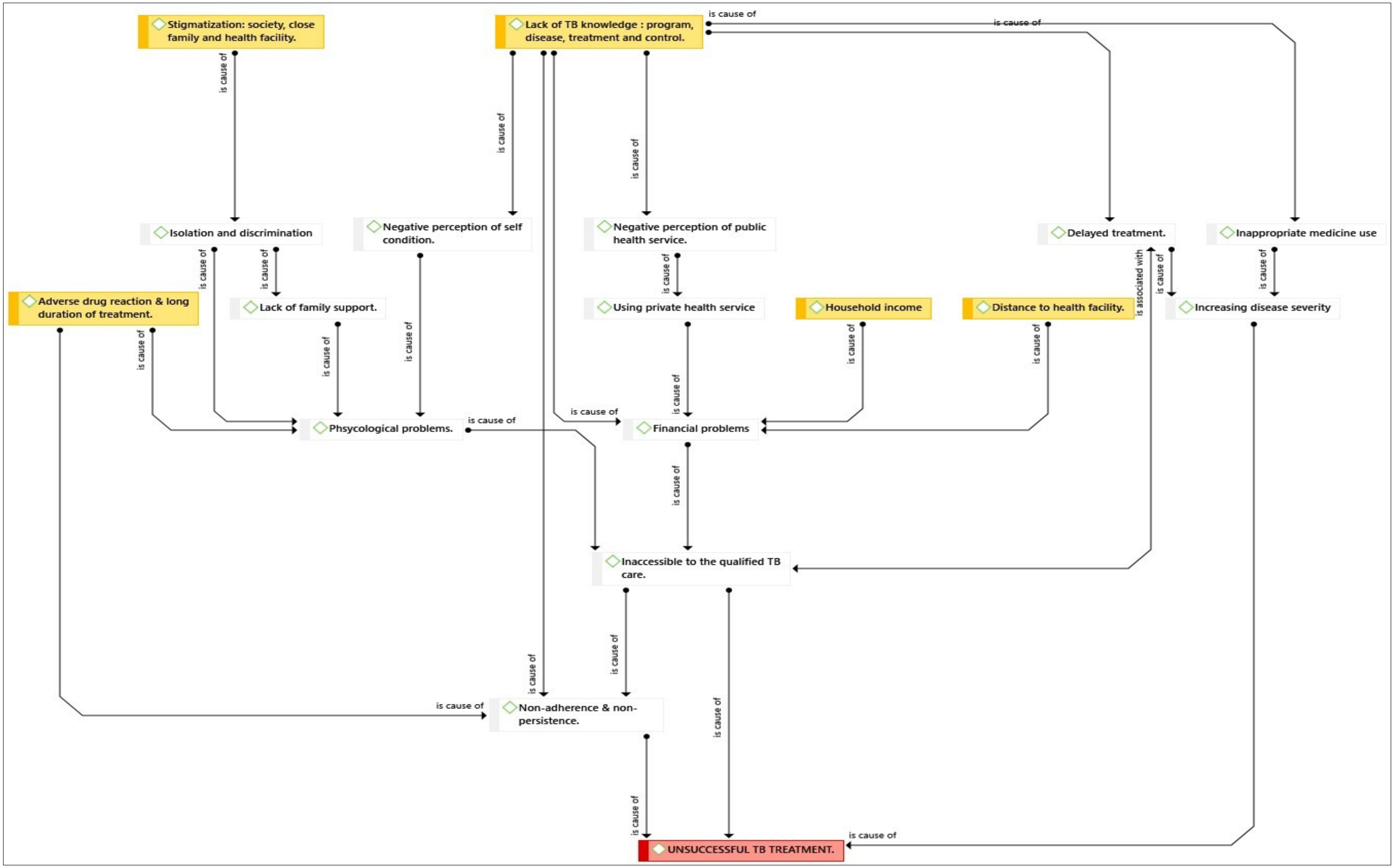

Additional file 5. The constructed barriers to successful tuberculosis treatment from the patient perspectives
